# Pediatric-Onset Epilepsy and Developmental Epileptic Encephalopathies Followed by Early-Onset Parkinsonism

**DOI:** 10.3390/ijms24043796

**Published:** 2023-02-14

**Authors:** Carlotta Spagnoli, Carlo Fusco, Francesco Pisani

**Affiliations:** 1 Child Neurology and Psychiatry Unit, Department of Pediatrics, Presidio Ospedaliero Santa Maria Nuova, AUSL-IRCCS di Reggio Emilia, 42122 Reggio Emilia, Italy; 2 Human Neurosciences Department, Sapienza University of Rome, 00185 Rome, Italy

**Keywords:** early-onset Parkinsonism, epilepsy, genetics, developmental and epileptic encephalopathies

## Abstract

Genetic early-onset Parkinsonism is unique due to frequent co-occurrence of hyperkinetic movement disorder(s) (MD), or additional neurological of systemic findings, including epilepsy in up to 10–15% of cases. Based on both the classification of Parkinsonism in children proposed by Leuzzi and coworkers and the 2017 ILAE epilepsies classification, we performed a literature review in PubMed. A few discrete presentations can be identified: Parkinsonism as a late manifestation of complex neurodevelopmental disorders, characterized by developmental and epileptic encephalopathies (DE-EE), with multiple, refractory seizure types and severely abnormal EEG characteristics, with or without preceding hyperkinetic MD; Parkinsonism in the context of syndromic conditions with unspecific reduced seizure threshold in infancy and childhood; neurodegenerative conditions with brain iron accumulation, in which childhood DE-EE is followed by neurodegeneration; and finally, monogenic juvenile Parkinsonism, in which a subset of patients with intellectual disability or developmental delay (ID/DD) develop hypokinetic MD between 10 and 30 years of age, following unspecific, usually well-controlled, childhood epilepsy. This emerging group of genetic conditions leading to epilepsy or DE-EE in childhood followed by juvenile Parkinsonism highlights the need for careful long-term follow-up, especially in the context of ID/DD, in order to readily identify individuals at increased risk of later Parkinsonism.

## 1. Introduction

Parkinsonism is the primary type of hypokinetic movement disorder (MD), sometimes also referred to as hypokinetic-rigid or akinetic-rigid syndrome. Early-onset Parkinsonism is defined as onset of Parkinsonism at ≤40 years, juvenile Parkinsonism as the onset < 21 years and young-onset Parkinsonism as the onset between 21 and 40 years of age [1]. While the incidence of Parkinsonism has been estimated as 0.8 per 100,000 person-years in the 0–29 y age group, it raises to 3.0 per 100,000 person-years in the 30–49 y age group [2]. As a comparison, the incidence of Parkinson’s disease (PD) is 0.04% in the 40–49 y age group and 1.1% in the 70–79 y age group [3].

Early-onset Parkinsonism is etiologically different from Parkinson’s Disease (PD). PD is a multisystemic synucleinopathy, whose neuropathological hallmark is the selective loss of dopaminergic neurons in the pars compacta of the substantia nigra, with intraneuronal proteinaceous cytoplasmic inclusions (Lewy body) deposition [4]. It is a common multi-factorial neurodegenerative disorder, in which various pathogenic factors lead to protein misfolding and aggregation, with premature neuronal death. These include genomic variants, epigenetic modifiers, environmental toxic factors, oxidative stress, neuroimmune/neuroinflammatory reactions, hypoxic-ischemic conditions, metabolic deficiencies, and ubiquitin–proteasome system dysfunction [5]. Additionally, pathogenic variants in a series of primary genes have been recognized as the cause of autosomal dominant (AD) and autosomal recessive (AR) forms of PD. Most defective loci identified in association with increased PD risk are involved in pathogenic pathways leading to premature neurodegeneration [synuclein accumulation, mitochondrial dysfunction, autophagic impairment, and oxidative and endoplasmic reticulum (ER) stress] [6]. The expression of PD-related pathogenic and susceptibility genes is regulated by the epigenetic machinery (DNA methylation, chromatin remodeling, histone modifications, and microRNA regulation), which may contribute to selective nigrostriatal dopaminergic injury [5].

Genetic early-onset Parkinsonism presenting through infancy to adolescence is also clinically different from PD, due to the frequent co-occurrence of a hyperkinetic disorder, additional neurological or systemic findings, and a wider picture of associated neuroimaging findings. An association between early-onset Parkinsonism and epilepsy is estimated in up to 10–15% of cases [7].

Epileptic encephalopathies (EE) are epilepsy syndromes in which the abundant epileptiform activity interferes with developmental trajectories, resulting in cognitive slowing or regression. However, in order to account the notion that in diverse severe genetic disorders, the genetic mutation in itself has detrimental developmental consequences, the term developmental encephalopathy (DE) has been proposed in the 2017 International League Against Epilepsy (ILAE) classification of epilepsies [8]. Genetic developmental and epileptic encephalopathies (DE-EE) largely represent complex neurodevelopmental disorders in which intellectual disability, developmental delay, behavioral or psychiatric issues, autistic features or additional neurological and extra-neurological features coexist with seizures [9]. The association with movement disorders (MD) has been recognized in a growing number of these complex neurodevelopmental disorders. In the majority of cases, infants and children develop hyperkinetic movement disorders, while hypokinetic forms are much rarer [10], but possibly underestimated. Furthermore, the association between epilepsy and hypokinetic disorders, even outside of the DE-EE spectrum, has possibly been the least investigated.

In this narrative review, the clinical and genetic landscape of pediatric-onset epilepsies and DE-EE with later juvenile or early-onset Parkinsonism will be described. 

## 2. Materials and Methods

We defined epilepsy and DE-EE following the latest ILAE classification published in 2017 [9]. 

Parkinsonism was defined by the presence of at least two cardinal features of PD (bradykinesia, muscular rigidity, 4–6 Hz resting tremor, postural instability) [1]. Early-onset Parkinsonism was defined as the onset of Parkinsonism under the age of 40, while juvenile Parkinsonism is defined as the onset under the age of 21 [1]. The classification of Parkinsonism in children follows the proposal by Leuzzi et al. distinguishing between developmental Parkinsonism; infantile and early childhood degenerative Parkinsonism; Parkinsonism in the setting of neurodevelopmental disorders; Parkinsonism in the setting of multisystemic brain diseases; juvenile Parkinsonism and dystonia-Parkinsonism; and acquired Parkinsonism [11].

The literature review was last performed on 31st October 2022 on PubMed [search terms: (genetic) AND (epilepsy) OR (epileptic encephalopathy) AND (early-onset) OR (juvenile) AND (Parkinsonism)]. The included article types (as per PubMed filtering) are case reports, “classical articles”, clinical studies, clinical trials, clinical trials phases I-IV, editorials, letters, meta-analyses, observational studies, and randomized controlled trials. Pertinent reviews and systematic reviews were manually checked, in order to establish if they provided additional sources of clinical and molecular data on the desired phenotypes. Papers were included if they provided a definite diagnosis and clinical information on the neurological phenotype (i.e., age of epilepsy and Parkinsonism onset, neurological examination, and seizure types).

**Figure 1:**
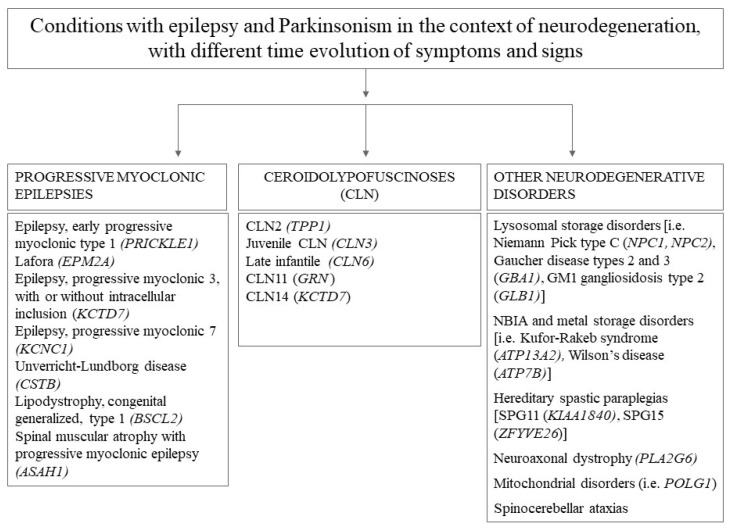
Excluded neurogenic conditions featuring epilepsy and Parkinsonism in the context of neurodegeneration.

Articles were considered as “non-pertinent” if they described neuropathological, epidemiological, therapeutic, neuroradiological or basic science/molecular biology results, or patients presenting with a phenotype or an etiology not corresponding to the subject of this review (i.e., progressive myoclonic epilepsies, inborn errors of metabolism, and autoimmune disorders). A short list of excluded neurogenic conditions possibly associating Parkinsonism and epilepsy in the context of neurodegeneration is summarized in Figure 1. A detailed and comprehensive list of causes of Parkinsonism in children is available in [11].

**Figure 2:**
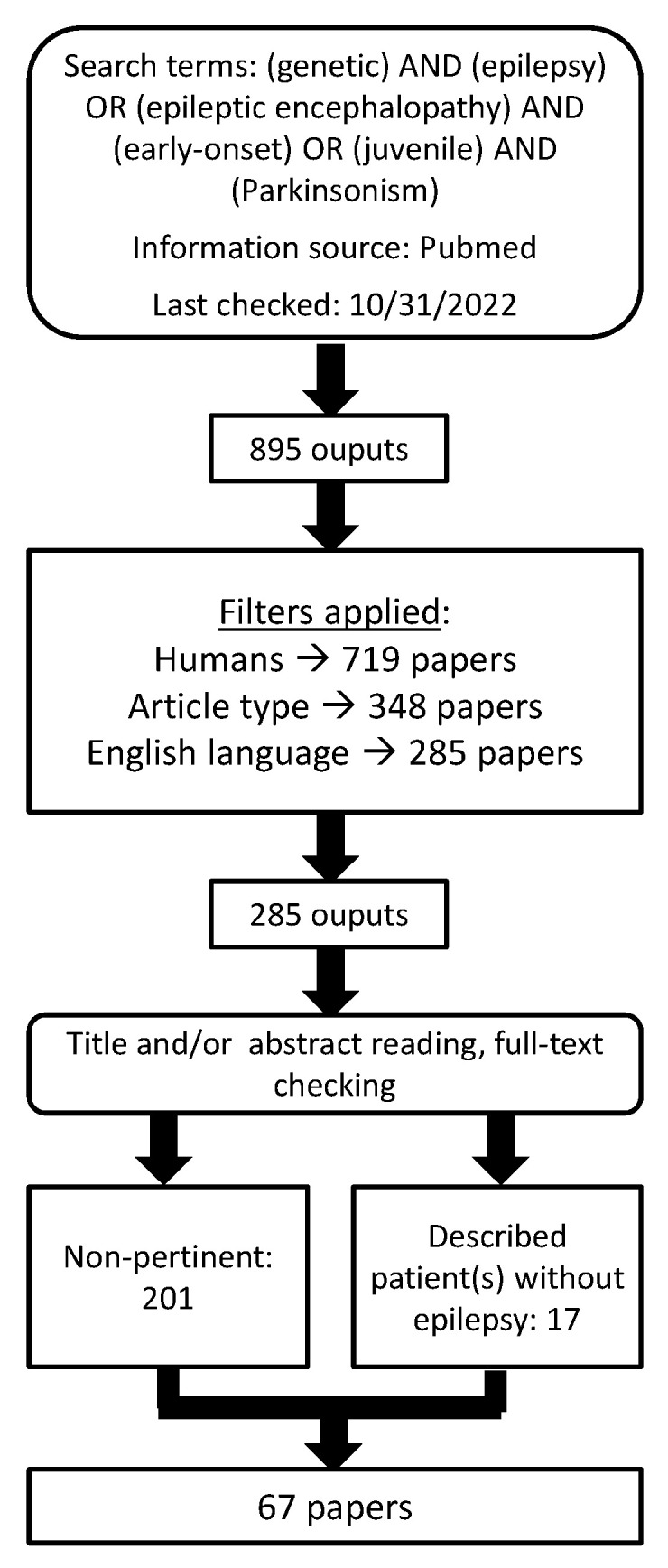
Flow chart depicting article selection process.

Finally, further papers were retrieved by checking papers’ references lists, and by reading relevant review papers on related topics. The selection process has been depicted in a flow chart (Figure 2).

## 3. Results

**Figure 3:**
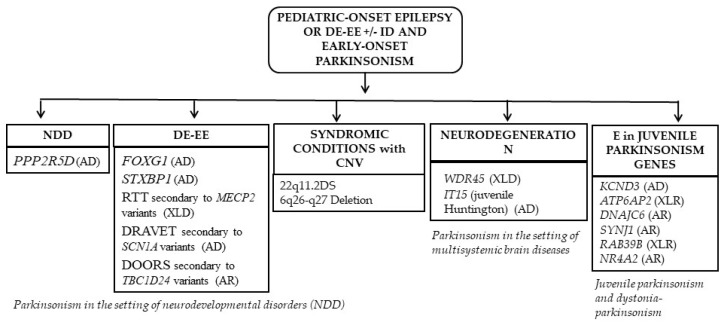
Classification and overview of the disorders described in this review. List of abbreviations: AD: autosomal dominant, AR: autosomal recessive, *ATP6AP2*: ATPase H+ transporting accessory protein 2, CNVs: copy number variations, DE-EE: developmental and epileptic encephalopathies, *DNAJC6*: DnaJ heat shock protein family (Hsp40) member C6, DS: deletion syndrome, E: epilepsies, *FOXG1*: forkhead box G1, ID: intellectual disability, IT15: huntingtin, *KCND3*: Potassium Voltage-Gated Channel Subfamily D Member 3, *MECP2*: methyl-CpG binding protein 2, NDD neurodevelopmental disorder, *NR4A2*: Nuclear Receptor Subfamily 4 Group A Member 2, *PPP2R5D*: Protein Phosphatase 2 Regulatory Subunit B’Delta, *RAB39B*: Member RAS Oncogene Family, RTT: Rett syndrome, *SCN1A*: sodium voltage-gated channel alpha subunit 1, *STXBP1*: syntaxin binding protein 1, *SYNJ1*: synaptojanin 1, *TBC1D24*: TBC1 domain family member 24, *WDR45*: WD repeat domain 45, XLD: X-linked dominant, XLR: X-linked recessive.

Genetic conditions characterized by the development of an early-onset Parkinsonism following epilepsy or DE-EE will be presented according to the following scheme: Parkinsonism in the setting of neurodevelopmental disorders including DE-EE; syndromic conditions with copy number variations (CNVs) or chromosomopathies; neurodegeneration with brain calcification genes; and juvenile Parkinsonism genes (Figure 3).

### 3.1. Neurodevelopmental Disorders and DE-EE (Table 1)

#### 3.1.1. *PPP2R5D* (Protein Phosphatase 2 Regulatory Subunit B’Delta)

The protein phosphatase type 2A (PP2A) is a heterotrimeric ubiquitous serine/threonine phosphatase, composed of a scaffolding subunit A, a regulatory subunit B, and a catalytic domain C [12]. *PPP2R5D* encodes the B56δ isoform of the subunit B, which is highly expressed in adult brain [12].

Less than 30 individuals mainly harboring recurrent, de novo missense variants were reported with *PPP2R5D*-related neurodevelopmental disorder, a rare autosomal-dominant disorder featuring developmental delay and intellectual disability (Intellectual developmental disorder, autosomal dominant 35, MRD35, Mendelian Inheritance in Men number MIM#616355). Additional clinical presentations include hypotonia, seizures, autism spectrum disorder, speech impairment, macrocephaly, facial dysmorphism and variable skeletal, endocrine and cardiac defects [13]. Parkinsonism with onset between 20 and 40 years in the context of stable intellectual disability during childhood was reported in four adults carrying the same p.(Glu200Lys) variant, none of whom suffered from epilepsy [14].

Subsequently, one female patient, carrying the de novo missense variant c.592G > A, p.(Glu198Lys) was described, presenting with hypotonia, severe developmental delay, drug responsive seizures with onset at 1 year of age, progressive hypertonia and dystonia and a decline of motor abilities since early adulthood, with stable cognitive functioning [13].

Neuropathologic findings from one patient [14] show focal substantia nigra atrophy and absence of Lewy body pathology, reminiscent of [15] or 1-methyl-4-phenyl-1,2,3,6-tetrahydropyridine-related Parkinsonism [16], and, thus, analogously suggest primary mitochondrial impairment and oxidative stress [16,17].

#### 3.1.2. *FOXG1* (Forkhead Box G1)

*FOXG1* acts as a transcription repressor. It plays an important role in fetal telencephalon development [18] and it is a key regulator in the development and territorial specification of the anterior brain, promoting neocortical lamination, neurogenesis, and dendritogenesis [19]. In the human adult brain, it is highly expressed in the basal ganglia (especially in the putamen and caudate) [20]. A role in regulating neuronal death has been postulated [21,22].

*FOXG1*-related syndrome is a neurodevelopmental disorder (NDD) characterized by severe postnatal microcephaly, developmental delay (DD), developmental encephalopathy (DE), hyperkinetic MDs, absent language, autistic features, and severe cognitive impairment [23,24]. The typical MD features chorea/athethosis, orolingual/facial dyskinesias, dystonia, but also stereotypies and non-epileptic myoclonus, mixed in more than 90% of patients [24]. Abnormal gyration, including frontal pachygyria, mildly/moderate simplified gyration, corpus callosum hypogenesis (mainly in its frontal part), and moderate-to-severe myelination delay (improving or normalizing with age) are often identified on neuroimaging [23]. Isolated reports of genetically confirmed cases followed-up until adulthood document an adolescence-to-adulthood-onset of hypokinetic MD [19,25].

One patient harboring the c. 250delC, p.(Gln86Argfs*106) in the N-terminal domain followed up until 17.3 years of age had early-onset (<1 year of life) hyperkinetic MD with predominantly orofacial dyskinesia, followed by stereotypies (mainly hand wringing) and choreo-athethosis since early childhood, decreasing with age. In adolescence, a hypokinetic MD with prominent dystonia and rigidity developed over trunk and extremities [19]. Uneven cognitive abilities in terms of verbal and non-verbal cognitive domains were also documented, gross motor skills and expressive language being the most severely affected [19]. One female patient was followed-up until 38 years. She had severe DD (walking at 5 years, non-verbal), and microcephaly. She had drug-responsive epilepsy characterized by bilateral tonic–clonic seizures. She showed stereotypies since childhood (frequent rubbing/twiddling hand movements and occasionally trunk rocking). At around 30 years of age, dyskinetic MD began and walking difficulties worsened. Aged 38, her neurological examination was significant for generalized dystonia with predominant craniofacial involvement, athethoid movements of her four limbs, and akinetic-rigid Parkinsonism without tremor. Her brain MRI showed a simplified gyral pattern and reduced white matter volume. She carried the c.610C > T, p.(Leu204Phe) variant. Dopamine transporter imaging with Iodine 123-radiolabeled 2β-carbomethoxy-3β-(4-iodophenyl)-*N*-(3-fluoropropyl) nortropane (^123^I-FP-CIT) or Dopamine Transporter Scan (DaTSCAN) showed moderate asymmetrical reduction in striatal uptake, predominating in the putamina [25].

#### 3.1.3. *STXBP1* (Syntaxin-Binding Protein 1)

Pathogenic variants in *STXBP1*, a gene involved in the exocytosis of synaptic vesicles, cause early infantile epileptic encephalopathy type 4 (EIEE4), or infantile spasms (IS) with non-syndromic encephalopathy, and frequent co-occurrence of MD [26]. Seizures are frequently drug-resistant. Brain magnetic resonance imaging (MRI) findings include cerebral atrophy, abnormal myelination, and hypoplasia of the corpus callosum [26,27,28]. Progression of neurologic symptoms has been reported in older patients, with onset of extrapyramidal features reminiscent of PD since teenage years. A 19-year-old woman with normal development until 2 years of age, a febrile seizure at 6 months, suffered from drug-refractory generalized epilepsy from the age of 6 and developed stimulus-sensitive myoclonus from the age of 13, and bradykinesia, antecollis, mood swings, impulsivity, heteroaggressive behavior, and visual hallucinations from the age of 18. She harbored the de novo p.Arg406His variant in the *STXBP1* gene. Good seizure and motor response to cannabidiol was reported [29].

A patient with the de novo *STXBP1* c.416C > T, p.(P139L) missense pathogenic variant in exon 6 presented with neonatal-onset epilepsy and by age 6 weeks showed a mixture of tonic and intermittent focal seizures controlled with phenobarbital by 4 months, global DD, ataxia by 6 years, head nodding stereotypies by age 7, dystonic posturing of her legs and a prominent dystonic tremor of her upper limbs at age 9. By age 12, an overt extra-pyramidal syndrome (resting tremor, cogwheel rigidity, and hypomimia) with pyramidal features (global hyperreflexia, upgoing plantars) developed. Her muscle biopsy showed profound deficiency in complex I of the mitochondrial respiratory chain [30].

A recent observational study performed on 30 adult carriers of *STXBP1*-related DE-EE, of whom 19 with available video tapes, documented hypomimia in 8/30 (27%), bradykinesia in 3/30 (10%), and rigidity in 2/30 (7%), reported as cogwheel rigidity in one. Non-specified ataxia was present in eight patients. Of note, the authors underlined a high occurrence of periods of regression, occurring into adulthood [31].

The suggested patho-mechanism for missense variants in *STXBP1* genes is haploinsufficiency through destabilization of the native folded state of the protein [32]. Pathogenic *STXBP1* variants disrupt neuronal function by affecting vesicular fusion, by altering cortical development, by affecting neuritogenesis and by inducing neurodegeneration. Defective endocytosis causes impaired synaptic transmission. Neuronal development is disrupted by the negative effect of defective exocytosis on dendrite growth, explaining the complex NDD with EE. Neurites from neurons expressing variants causing *STXBP1*-EE also exhibit signs of neurodegeneration, and in some cases ring-like structures reminiscent of Lewy bodies [32]. 

#### 3.1.4. Rett Syndrome Secondary to methyl CpG Binding Protein 2 (*MECP2*) Variants

During the first 10–20 years of life, many Rett patients demonstrate gradual evolution from generalized hypotonia to progressive muscle rigidity, often accompanied by dystonic posturing and progressive scoliosis, typically paralleled by declining gross motor abilities (“late motor deterioration stage”), during which, in contrast, social skills often improve [33]. According to previous reports, rigidity can be found in 48–84% [34,35] of cases. Age of onset has been exceptionally reported as early as 3 years of age [35], although its prevalence increases with age, to be present in all participants aged ≥13 years according to [35]. Ankle rigidity is reported to appear first, followed by proximal legs, arms, the neck, and face. Furthermore, teenagers and adults with more severe Rett phenotypes evolve into disabling generalized parkinsonian state with marked rigidity, hypomimia, and generalized bradykinesia/akinesia [34,36]. Partial or profound postural instability is also pervasive [35]. Bradykinesia is usually difficult to quantify, due to abundant hand stereotypies, but tends to be prominent in older individuals with generalized rigidity. Hand and foot stereotypies are also barriers to correctly quantifying resting tremor [35]. According to some studies, Rett rigidity distribution scores tend to be lower in ambulatory and in verbal patients, with no statistically significant differences according to gene variant type [35]. However, according to some authors, higher percentages of patients with truncating mutations were non-ambulatory in comparison with those having missense mutations [35]. In a prospective study of gross motor abilities in 70 ambulatory or partially ambulatory participants with Rett in the Australian Rett Syndrome Database, gross motor skills declined slightly during the 3–4 years follow-up in 52.9% (versus 31.4% improving slightly, and 10.0% being stable) [37]. 

Evidence is emerging to suggest that one important factor in the pathogenesis of parkinsonian features in Rett may be a dysfunction in the nigrostriatal dopaminergic projection system [38,39]. Limited pathological studies show that substantia nigra pars compacta neurons are smaller and more hypopigmented than normal [40,41]. There are conflicting reports concerning dopamine metabolite levels in cerebrospinal fluid (CSF) of Rett patients [40,41,42,43]. A study on 64 participants showed that average homovanillic acid (HVA) and 5-hydroxyindoleacetic acid (5-HIAA) levels were significantly lower than in controls. Twelve of 64 (19%) of the Rett participants had CSF HVA levels below normal and 15/64 (23%) had 5-HIAA levels below normal [44]. 

Brain serotonin and norepinephrine levels are also significantly reduced in mecp2 knockout mice. The Rett mouse model has lower brain dopamine levels than wild-type mice and marked impairments in movement speed and balance [44]. Early identification of low CSF HVA levels in Rett patients has been proposed as a clue to the later onset of parkinsonian features [35], even if, while Rett rigidity scores correlate negatively with CSF HVA levels, they do not with 5-HIAA acid levels [35].

#### 3.1.5. Dravet Syndrome Due to Sodium Voltage-Gated Channel Alpha Subunit 1 (*SCN1A*) Variants

Children under six years of age have a normal or variable gait pattern, possibly with joint hypermobility and ataxic features [45,46]. By adolescence, some patients develop a flexed gait pattern with passive knee extension deficit and bony malalignment: crouch gait [45]. Parkinsonian gait and extrapyramidal signs become evident in adulthood [47,48] and it is estimated that 25–30% of adults with Dravet syndrome (DS) have extrapyramidal features. Other neurological signs, such as spasticity, dysarthria, and intentional tremor are infrequent [49]. Parkinsonian features were present in at least 11/14 *SCN1A*-positive DS cases [47,48]. In detail, antecollis was present in 9/14, and parkinsonian gait in 8/14 [48]. The patho-mechanism for this evolution is possibly linked to *SCN1A* gene expression in the basal ganglia [50]. The vulnerability of the dopaminergic system to ageing [51] might explain why Parkinsonism was only described in adults and correlates with age [47].

#### 3.1.6. TBC1 Domain Family Member 24 (*TBC1D24*)

Pathogenic variants in the *TBC1D24* gene have been implicated in different human diseases, including non-syndromic deafness (both AR and AD), onychodystrophy, osteodystrophy, mental retardation, and seizures (DOORS) syndrome; and infantile-onset epilepsy, spanning from benign self-limiting, such as familial infantile myoclonic epilepsy (FIME), to severe early onset epileptic encephalopathy with early death (early-infantile epileptic encephalopathy type 16, EIEE16) and progressive myoclonus epilepsy (PME) [52], infantile-onset paroxysmal dyskinesia and ataxia [53], and exercise-induced paroxysmal dystonia [54]. The range of associated seizure types is wide, including infantile spasms, febrile seizures, tonic, absence, tonic–clonic and focal, both with retained and impaired awareness, although clonic or myoclonic seizures (often occurring in prolonged clusters) predominate. The majority of patients suffer from drug-resistant epilepsy. Brain MRI most often reveals cerebellar and/or cerebral atrophy, but delayed myelination or vermian hypoplasia have also been described [52].

In the widest published cohort, 7/48 patients were ataxic, while 8/48 patients had extrapyramidal symptoms, including supranuclear gaze palsy, dystonia, tremor, dyskinetic movements with upper limb dystonia, and progressive deterioration of gait. Of note, one patient had Parkinsonism since the age of 21 (mixed resting-postural tremor and rigidity of the right arm and right-side bradykinesia; mild dysdiadochokinesia and reduced tendon reflexes) [52]. One additional report describes myoclonic epilepsy, cerebellar ataxia, cognitive impairment, Parkinsonism, photosensitivity and psychosis. Notably, while tremor and focal dystonic attacks began at 7 months, this patient had a first generalized seizure at 11 months. At the same age, she acquired independent walking, although broad-based. Her brain MRI at 2.5 years of age showed cerebellar atrophy [55]. 

The *TBC1D24* gene is involved in regulation of synaptic vesicle trafficking and in brain and somatic development. It interacts with the adenosine diphosphate (ADP) ribosylation factor 6 (ARF6), a guanosine triphosphate (GTP)-binding protein involved in membrane exchange between plasma membrane and endocytic compartments [56,57]. Interestingly, it also contains a TBC, LysM, Domain catalytic (TLDc) domain, involved in oxidative stress resistance [58].

#### 3.1.7. *FRRS1L*

Ferric chelate reductase 1 like *(FRRS1L)* encodes an aminomethylphosphonic acid, α-amino-3-hydroxy-5-methyl-4-isoxazolepropionic acid (AMPA) receptor (AMPAR) outer-core protein. Loss of *FRRS1L* function affects AMPAR constituency and AMPAR function, implicating chronic abnormalities of glutamatergic neurotransmission [59].

*FRRS1L*-related encephalopathy (Early Infantile Epileptic Encephalopathy-37) is a rare DEE so far described in 15 children from six families [59,60,61], all presenting with epilepsy, MDs and severe DD or regression. Seizure types include clonic, hemi-clonic, bilateral tonic–clonic, spasms and ‘multifocal’ seizures. They tend to be refractory. Specific and detailed electroencephalographic (EEG) patterns have been reported in a few patients, including two with continuous spikes-and-waves during slow sleep (CSWS) [61,62] and one with occipital spike waves with fixation off sensitivity [62]. Eight patients from four families were described in a first report, with encephalopathy, epilepsy, and progressive choreoathetosis, harboring biallelic pathogenic loss-of-function variants in the *FRRS1L* gene. All had normal early development, but the majority (6/8) regressed at a mean of 18 months (range: 4–24 months), ceasing to walk and losing expressive language. This coincided with epilepsy onset (featuring hemiclonic and tonic–clonic seizures), which showed some response to carbamazepine and/or valproate. Patients’ neurological examination was significant for global DD, diffuse hypotonia, and generalized chorea with paucity or lack of volitional movements in 7/8. Additionally, four had pyramidal signs. Brain MRI was initially normal, although repeat neuroimaging demonstrated cortical and cerebellar atrophy and flattening of the caudate heads. Of note, environmental response declined in all cases and in late adolescence their hyperkinetic MD was substituted by a rigid-akinetic state [59]. All subsequently published patients were younger when described and no follow-up data are available [61,62].

AMPA-type glutamate receptors (AMPARs) operate on fast excitatory neurotransmission, which is fundamental for normal brain function and mediates a huge part of the excitatory post-synaptic currents (EPSC), which promote synapses formation and maturation, trigger activity-dependent processes, and result in activity-initiated plasticity, an important factor for learning and memory. FRRS1L [together with carnitine palmitoyltransferase 1 (CPT1c)] is a key component of distinct assemblies of AMPARs, localizing to the ER and very important in AMPARs biogenesis, as all surface AMPARs emerge from ER-located assemblies. In mice, Frrsl1 deletion results in increased proportion of immature AMPA receptors, leading to cytoplasmic retention and reduced specific AMPA receptor levels in the post-synaptic membrane [63]. Decreased EPSC amplitudes in excitatory glutamatergic synapses is likely to negatively influence both synaptogenesis and signal transmission and, as a result, synaptic plasticity [64]. Additionally, through imbalance between excitatory and inhibitory synaptic transmission, these detrimental changes are thought to facilitate seizures [65,66]. 

A proposed explanation for regression could be FRRS1L upregulation during postnatal development [67]. In embryonic mice, FRRS1L is expressed in the forebrain [59]. Developmentally, it might play a role in neuronal maturation, through AMPARs action in promoting synaptic formation and maturation [68]. In the adult mouse brain, its expression is located in excitatory neurons in the cerebral cortex, hippocampus and midbrain, medium spiny neurons in the striatum, granule cells in the dentate gyrus, and Purkinje cells in the cerebellum [69]. 

**Table 1:** Neurodevelopmental disorders and DE-EE with Parkinsonism.

Gene/Condition Name	Inheritance	Cognition	Additional NDD Features	Neurological Examination and MDs	Syndromic Features	Epilepsy	Parkinsonism, Onset Age
*PPP2R5D*	AD	DD, ID	ASD, speech impairment	Infantile hypotonia, progressive hypertonia	Macrocephaly, facial dysmorphism, variable skeletal, endocrine, and cardiac defects	Possible	20–40 y
*FOXG1*	AD	DD, severe cognitive impairment	Absent language, autistic features	Hyperkinetic MDs	severe postnatal microcephaly	DE	Teenage years/adulthood
*STXBP1*	AD	Severe DD, ID	ASD	Hyperkinetic MD	/	DE-EE	Teenage years
Rett Syndrome *MECP2*	XL	Absent/unremarkable before regression phase	Regression of motor and language function	Hypotonia, hyperkinetic MD	Acquired microcephaly, progressive scoliosis	Non-specific epilepsy or DE-EE	Teenage years/adulthood
Dravet Syndrome *(SCN1A)*	AD	Variable DD/ID	Possible autistic features	Initially normal, then ataxia, spasticity, dysarthria, crouch gait	Clinical criteria for Rett syndrome diagnosis	DE-EE	Adulthood
*TBC1D24*	AD	DD, ID	/	Chronic and paroxysmal hyperkinetic MD	Possible cerebro-cerebellar malformation	Usually drug-resistant	Early 20′s
*FRRS1L*	AD	Normal early development followed by regression with GDD	Additional NDD features	Diffuse hypotonia, chorea, pyramidal signs	/	Multiple seizure types, mainly clonic/myoclonic	Late adolescence

#### 3.1.8. Syndromic Conditions with CNVS

##### 22q11.2 Deletion Syndrome

22q11.2 deletion syndrome (OMIM #192430, #188400) affects 1:4000 live births. Nearly 90% of patients carry a 3 Megabases (Mb) deletion including at least 52 known genes, while 8% of cases carry a nested 1.5 Mb deletion including at least 31 genes [70]. Its phenotypic expression, although variable, includes DD, intellectual disability (ID) of varying degrees, palatal anomalies (i.e., velopharyngeal insufficiency causing hypernasal speech), heart malformations, calcium metabolism impairment with hypocalcemia, and subtle dysmorphic features. Neurological features include susceptibility to seizures, either provoked by hypocalcemia or fever, or epilepsy, both generalized (juvenile myoclonic epilepsy or other generalized genetic epilepsies, such as absences, or generalized tonic–clonic) or focal (structural, secondary to polymicrogyria, subependymal nodular heterotopia or focal cortical dysplasia, or non-structural). Psychiatric comorbidities are also frequent, including schizophrenia spectrum disorders, anxiety, depression, autism spectrum disorder (ASD), and attention deficit hyperactivity disorder (ADHD). The role of 22q11.2 deletion syndrome as a risk factor for PD has been increasingly recognized [71,72,73,74,75,76,77]. In a Canadian study, in contrast with the general population estimate of 22q11.2 deletion syndrome as 1 in 4000 (0.025%), in an early PD cohort it was 1 in 224 (0.4%) [71]. A further international study on the role of 22q11.2 deletion syndrome as a risk factor for idiopathic PD documented a prevalence of 22q11.2 deletion syndrome of 0.49% in patients with PD onset < 45 years and of 0.04% in patients with onset at ≥45 years [77], highlighting the role of this deletion as a risk factor for early-onset Parkinsonism. The estimated prevalence of early-onset PD in 22q11.2 deletion syndrome is 0.028 per 100.000 people [77]. Among 56 adult Italian patients, with a mean age of 29 years, 31 (55%, 16 of whom on antipsychotics) were diagnosed with Parkinsonism. Asymmetric motor involvement was more common in patients not receiving antipsychotics, while action tremor and axial involvement were more common in patients on antipsychotic medications. The authors also found a correlation between psychosis and lower intellectual quotient (IQ) and epilepsy. None of their patients with parkinsonian features had epilepsy [78]. Careful monitoring is essential for a prompt diagnosis of Parkinsonism, especially in patients with psychosis, to avoid diagnostic delay (6–10 years versus < 1 year of symptoms onset) [71].

In post-mortem pathology of three cases with 22q11.2 deletion syndrome and a clinical diagnosis of early-onset Parkinsonism, Lewy bodies were detected in 2/3 cases, while the third showed extensive neuronal loss in the substantia nigra pars compacta, along with neuronal loss in the locus ceruleus and the dorsal motor nucleus of the 10th cranial nerve. Gliosis was also observed in the midline nuclei and the anterior and posterior group of the intralaminar nuclei, in the absence of a-synuclein pathology. This variable presence of a-synuclein aggregation is similar to findings in leucine rich repeat kinase 2 (*LRRK2*)-related PD [71]. Reduced striatal binding on dopamine transporter DAT scan imaging was also documented [77,79]. 

According to [71], the proximal 22q11.2 deletion region shared by individuals developing PD contains plausible candidate genes, such as microRNA miR-185, predicted to target LRRK2 [80] and DiGeorge Critical Region 8 (DGCR8), a key gene in the biogenesis of brain microRNA [81]. Other possible candidate genes include Septin-5 (*SEPT5*), encoding a protein functionally interacting with Catechol-O-methyltransferase (COMT), essential for dopamine levels regulation, and six mitochondrial genes [82], all expressed in the brain and, when examined, showing gene dosage effects in 22q11.2 deletion mouse models [83]. Disruption of microRNA-mediated post-transcriptional regulation of gene expression in 22q11.2DS might affect the expression of PD risk genes elsewhere in the genome [84]. As PD only affects a minority of 22q11.2 deletion syndrome patients, other modifying factors might play a role [77]. Based on a whole genome study of 22q11.2 deletion syndrome patients with and without PD, the burden of rare deleterious missense variants was not found to be different. However, evaluation of a set of candidate genes for PD (excluding those included in the 22 q11.2 deletion syndrome region and known PD- related genes) documented a greater burden in 22q11.2 DS patients with PD, including rare variants in Kruppel-like factor 1J (*KLF1J*), a regulator of the monoamine oxidase B expression, and microtubule associated protein 2 (*MAP2*), a cytoskeleton protein found in Lewy bodies. The same variant affecting leucyl-tRNA synthetase 2, mitochondrial *(LARS2*), encoding for a mitochondrial aminoacyl-tRNA synthetase, which is downregulated in the dopaminergic neurons of the substantia nigra in PD, was detected in 2/3 patients, while two different variants were documented in 2/3 patients in the titin (*TTN*) gene. However, none was found in all of the three patients. Therefore, the authors proposed a “multi hit” hypothesis, in which hemizygosity of the 22q11.2 deletion region together with each individual’s cumulative genome-wide burden of rare deleterious PD-related variants leads to expression of PD in 22q11.2 deletion syndrome. The presence of a recessive allele on the intact 22q11.2 chromosome is considered less likely [85].

#### 3.1.9. 6q26-q27 Deletion

A recent systematic literature review documented 36 patients harboring deletions of 6q26-q2. The most frequent clinical features include ID/DD in 26/32 (81%), brain abnormalities in 23/32 (72%), facial dysmorphism in 21/32 (66%), hypotonia in 20/32 (63%), learning difficulty or language delay in 16/32 (50%). A total of 15/32 (47%) patients have drug-responsive seizure. Ictal semiology was described only in one patient, as focal impaired awareness with occasional evolution towards bilateral tonic–clonic.

Additional features include vertebral or spinal cord malformations, microcephaly, hydrocephalus, joint laxity, ventricular septal defect, ADHD, and autism spectrum disorder (6%, 2 out of 32), as well as hearing loss, and poor vision. Early-onset PD was reported in one patient (3%) [86]. One proximal critical region was identified at 6q26 and contains the *PRKN* gene, causative of juvenile PD type 2 (OMIM# 602544). The Parkin RBR E3 Ubiquitin Protein Ligase (*PRKN*) gene encodes a Really Interesting New Gene (RING) domain-containing E3 ubiquitin ligase involved in proteasome-dependent protein degradation. It also plays an important role in mitophagy and autophagy [87].

#### 3.1.10. Chromosomopathies

Parkinsonism in the context of dementia was reported in 6–9% of patients with Down syndrome (DS) [88]. Decreased dopamine concentrations were documented in the caudate and putamina of older DS patients, but data on Lewy body pathology are inconsistent. One single case report exists with early-onset Parkinsonism in a Klinefelter patient aged 27 years [89]. Klinefelter syndrome has been associated with epilepsy in 3/17 studied cases in [90] and in a single case report of absence epilepsy [91]. Additional, single cases of early-onset Parkinsonism are available for partial 6q syndrome (aged 35 years) [92] and partial 4q syndrome (aged 30 years) [93,94], although these two CNV syndromes do not seem to be associated with a significant increase in seizure risk (Table 2).

### 3.2. Neurodegeneration

#### 3.2.1. WD Repeat Domain 45 (WDR45)

*WDR45* is an important member of the WD repeat protein interacting with phosphoinositides, taking part in various biological processes, such as cell cycle, signal transduction, gene regulation, and apoptosis. Defects in autophagy are closely related to neurodegenerative disorders [95].

Its pathogenic variants cause neurodegeneration with brain iron accumulation (NBIA), a disease characterized by global DD and DE-EE in childhood, followed by regression in early adulthood (progressive dystonia, Parkinsonism, and dementia). Brain MRI shows iron accumulation in the substantia nigra (with a ‘halo’ of T1 hyperintensity) and globus pallidus [96]. Despite being an X-linked (XL) disorder, and being more frequent in females, clinical presentation is similar in both sexes, suggesting somatic mosaicism in surviving males and germline or somatic mutations in females, as well as skewing of X chromosome inactivation [95]. Epilepsy or DE/EE begins in infancy [97] or childhood [96], with refractory multiple seizure types, including epileptic spasms, tonic, focal non-motor with impaired awareness, myoclonic [98,99], atonic with head nodding, fever-sensitive, and bilateral tonic–clonic [96]. Sleep disorder, truncal hypotonia or spasticity can represent additional clinical elements in childhood [96]. Deterioration occurs from teenage years to the fourth decade. Parkinsonism is characterized by prominent bradykinesia, rigidity and freezing of gait, and a lower occurrence of tremor, while dystonia is common since adolescence or early adulthood, typically starting in the upper limbs. Although symptoms can improve with L-3,4-dioxyphenylalanine (L-DOPA), therapy is often discontinued due to side effects [96]. Cognitive deterioration parallels the onset of MD, with progressive loss of expressive language skills advancing to severe dementia.

Pathology is different from other NBIAs, as pathological changes in substantia nigra dominate those found in globus pallidus, with iron deposition and neuronal loss, axonal swelling, and gliosis. There is no “Lewy bodies” pathology, while diffuse tau pathology was demonstrated, with neuropil threads, pre-tangles, and neurofibrillary tangles [97,98]. Patients disclose reduced autophagic activity [97,98,99,100]. ER dysfunction with increased ER stress leading to neuronal apoptosis has been demonstrated in a mouse model [101,102].

#### 3.2.2. *IT15*

Huntingtin, the gene product of the *IT15* gene, is a large protein of 3144 amino acids, expressed in different organelles including the nucleus, endoplasmic reticulum, Golgi complex, and mitochondria. It normally contains 15–30 repeats of the triplet sequence CAG. Expansions with more than 40 repeats cause Huntington’s disease (HD), and expansions of more than 60 repeats are usually associated with Juvenile Huntington’s disease (JHD), defined by onset < 20 years of age. Onset < 5 years is exceptional [103]. Age at onset within JHD also correlates with the number of CAG repeats. Disease progression is more rapid, with reduced lifespan compared to adult-onset cases. In 80% of JHD cases, inheritance is from the father [104]. JHD accounts for 5–10% of all HD cases and shows important phenotypic differences from the adult form. Gait disturbance is more frequently a first presenting symptom in the high expansion subgroup, while loss of hand dexterity is more common in the low expansion subgroup [105]. Other researchers documented seizures or prominent oral motor dysfunction as the first symptoms [106,107]. 

Disease course is typically marked by gait, cognitive, and behavioral impairment and a slow but steady IQ decline, with regression in language skills, changes in academic performance, and inability to develop reading skills. The main behavioral issues include hyperactivity, opposition, and aggression [103]. Additionally, DD, severe gait impairment and epilepsy prevail in the high expansion group, while obsessive behavior is more common in the low expansion subgroup [105]. MDs includes dystonia and Parkinsonism (more prevalent in the high expansion subgroup), whereas ataxia is rarer and apparently less related to CAG repeats number. Oral dyskinesias are common. Chorea is generally absent to minimal. An akinetic-rigid syndrome occurs in 45–75% of patients [103,108,109,110,111].

The reported incidence of epilepsy in childhood-onset HD ranges from 27% to 50% [103,108,109]. Although seizures usually occur late in the disease course, they can sometimes occur at disease onset, usually paralleling the beginning of neurologic deterioration, more often in patients with high repeats number and in early-onset cases (<10 years). Generalized tonic–clonic are the most common seizure type followed by atypical absences and myoclonic seizures [103]. They are often refractory. There are no specific EEG characteristics, although generalized slow waves, multifocal spikes, polyspike waves, and bilateral synchronous spikes are often reported [112].

In all patients having neuroimaging, early, selective, bilateral and global involvement of both striatal nuclei with marked volume loss in the caudate nucleus and putamen, but cortical or white matter involvement is not typical [105] (Table 3).

### 3.3. Juvenile Parkinsonism Genes

#### 3.3.1. *KCND3*

The potassium voltage-gated channel subfamily D member 3 (*KCND3*) is a six trans-membrane segmented ion channel, involved in transient outward K+ current, with high expression in the central nervous system, especially in cerebellar Purkinje cells, deep nuclei, granule cells, and interneurons. 

Pathogenic loss-of-function variants in the *KCND3* gene cause the AD spinocerebellar ataxia (SCA) 19/22 [113,114], while gain-of-function variants cause cardiological disorders [115].

The clinical spectrum of neurological conditions associated with pathogenic *KCND3* variants has been extensively described in a recent review [116]. Age at onset can occur over the whole life span, and the main clinical presentation is ataxia, although onset with epilepsy or NDD (with or without ID) is also possible. During disease course, additional neurological symptoms and signs emerge, mainly a cerebellar syndrome, oculomotor abnormalities, cognitive impairment and MD (Parkinsonism, myoclonus, dystonia, and tremor). Peripheral neuropathy and pyramidal signs can develop. While late-onset cases are prominently ataxic, early-onset cases display a complex phenotype including ataxia, ID and epilepsy. In fact, this latter case only occurs in early-onset cases, affecting half of the patients and usually being drug-responsive, as only one patient (with EE) was found to be drug-resistant. Parkinsonian features, including rigidity or bradykinesia, are the most frequent MD (17% of late-onset and 20% of early-onset cases, respectively). Patients with early onset forms present with a NDD or epilepsy before the onset of cerebellar signs, while MDs can emerge later in the disease course [116].

#### 3.3.2. *ATP6AP2*

ATPase H+ Transporting Accessory Protein 2 (*ATP6AP2*) encodes an accessory unit of vacuolar ATPase (VATPase), a lysosomal enzyme expressed in different organs, including the brain. An XL parkinsonian syndrome with variable spasticity was first described in five men belonging to several generations within a single family. Two out of five had mild response to levodopa but one developed dystonic dyskinesias. They carried the synonymous mutation c.345C > T, leading to inefficient splicing of exon 4 [117]. Later, the description of seven men affected by epilepsy, and mild-to-moderate ID ensued. Epilepsy with tonic–clonic and atonic seizures began on average at 6.8 months of age (range: 4–14 months), and was followed in two cases by scholiosis and progressive gait disturbance with mild generalized rigidity and ataxia since the age of 8 [118]. Notably, a third patient was described [119], with ID, epilepsy with onset at 1 year of age with tonic–clonic seizures not responding to antiepileptic medications, and bradykinesia (without rigidity, rest tremor, or postural instability) when examined at 31 years of age. Their brain MRI showed global atrophy of the cerebral hemispheres, cerebellar atrophy and thinning of the corpus callosum. They carried the c.168 + 6T > A splice site mutation, presumably affecting splicing due to close proximity to the exon-intron boundary of exon 2. Although the two initially reported families [117,118] were considered to have two separate allelic disorders, in light of data coming from the third family, the current interpretation favors a single disorder with variable expressivity.

#### 3.3.3. *DNAJC6*

DnaJ Heat Shock Protein Family (Hsp40) Member C6 *(DNAJC6)* encodes the neuronal co-chaperone HSP40 auxilin, a neuron-specific clathrin-associated protein which is enriched in nerve terminals. Auxilin is the co-chaperone conferring specificity to the ATPase activity of its partner Hcs70 in clathrin uncoating. 

Juvenile Parkinsonism caused by the biallelic c.801-2 A > G variant in *DNAJC6* was first documented in two affected brothers with onset at 11 and 7 years of age, respectively. They had preserved cognition on a background of normal developmental milestones and unremarkable brain MRI findings. The variant results in the generation of two mis-spliced cDNA transcripts; an in-frame exon 7-skipped transcript lacking amino acids 268–328 and an out-of-frame transcript with an insertion of the last 91 nucleotides of intervening sequence (IVS) 6 (c.801 291) between exon 6 and 7, resulting in the addition of eight non-synonymous residues before reaching a termination codon. The normally spliced transcript was undetectable [120].

Three additional cases were later described, with generalized epilepsy (absence and tonic–clonic seizures) starting at 1–5 years of age, controlled on valproate. They had mild to moderate ID (IQ: 40–63), and early-onset (10–11 years) tremor, a shuffling gait and/or bradykinesia, and progressed to Parkinsonism (postural instability, resting and postural tremor, bradykinesia, rigidity) by the ages 13–22 years, intermittent dystonic symptoms and pyramidal signs, and, finally, anarthria and akinesia. All were wheelchair bound 10–15 years after disease onset. In one case, diffuse brain atrophy was detected. Response to L-Dopa, although good, was limited by severe side-effects [121]. 

A homozygous 80-kb deletion involving part of *DNAJC6* and leptin receptor (*LEPR*) genes was also identified in a 7-year-old boy with dysmorphic features, obesity, epilepsy (febrile seizures from 6 months of age, tonic–clonic seizures from 2.5 years of age, and CSWS from 5 years of age) and ID, but no signs of Parkinsonism, possibly depending on the patient’s age [122].

#### 3.3.4. *SYNJ1*

Synaptojanin *(SYNJ1*) encodes a brain-specific polyphosphoinositide phosphatase containing two consecutive phosphatase domains and playing an important role in late stages of clathrin-mediated endocytosis [123].

Two main phenotypes have been associated with *SYNJ1* pathogenic variants: a severe disorder with neonatal refractory epilepsy and neurodegenerative course (MIM #61738932) and early-onset Parkinsonism (MIM #61553033), with/without epilepsy [124]. The first phenotype is characterized by neonatal-onset epilepsy/infantile-onset EE evolving to multiple seizure types, profound ID, and neurodegenerative course (progressive spastic quadriplegia, dysphagia, and early death). Spectrophotometry of respiratory chain complexes showed either a combined deficiency in complex III and IV activities in liver and fibroblasts [124] or diminished levels of complex I [125]. A homozygous nonsense variant, expected to result in loss of protein formation, was found in a patient with early-onset intractable EE and tau pathology [125,126].

The second phenotype was described in four independent families showing Parkinsonism since the third decade of life. Half of them also had infrequent (or even single) bilateral tonic–clonic seizures with onset between infancy and adolescence [127,128,129,130]. The three initial families carried the same homozygous missense variant p.(Arg258Gln) located in the Sac1 domain [127], selectively abolishing the phosphatase function of this domain. Two siblings from a Tunisian consanguineous family carried the compound heterozygous variants p.(Leu1406Phefs*42) and p.(Lys1321Glu). The p.(Leu1406Phefs*42) variant disrupts the adaptor protein 2 (AP2) binding sites and disables synaptic and vesicle endocytic recycling in neurons. Both patients developed mild Parkinsonism at 16 and 21 years of age, respectively. One also had epilepsy with tonic–clonic seizures from 7 years of age. They showed good response to low-dose levodopa with no dyskinesia or motor fluctuations. They also had moderate cognitive impairment. Brain MRI was normal [130].

These two phenotypic presentations seem to correlate with residual phosphatase activity: variants leading to (nearly) complete loss of dual phosphatase activity lead to the first phenotype, while loss of dephosphorylation activity limited to the Sac1 domain leads to Parkinsonism with onset in early adulthood and mild increase in seizure susceptibility [124].

#### 3.3.5. *NR4A2*

Nuclear receptor subfamily 4 group A member 2, encoded by *NR4A2*, is a transcription factor involved in developing and maintaining dopamine-synthesizing cells. Its down-regulation severely alters dopaminergic pathways and motor control [131].

*NR4A2* haploinsufficiency was recently associated with an NDD with language delay and epilepsy [132]. Additional reports also disclosed its causative role in early-onset [133], including infantile-onset [134], dopa-responsive dystonia Parkinsonism. In one of these patients, epilepsy with bilateral tonic–clonic seizures was detected at 26 years of age and focal dystonia (initially paroxysmal) at 29, on a background of mild ID. This patient carried the c.326dupA, p.(Ser110Valfs*2) loss-of-function variant, which has also been described in a pediatric patient who presented during infancy with poor feeding, gastrointestinal symptoms, normal motor development and growth parameters, but delayed speech development and onset of seizures at 5 years of age. EEG background activity was normal, while interictal activity was characterized by high amplitude bilateral sharp waves in the centro-parieto-temporal region, significantly increasing during drowsiness and sleep (reminiscent of “rolandic” epilepsy). Sporadic focal motor seizures persisted for 2 years, usually during sleep, and were controlled by clobazam and sulthiame. Verbal comprehension and operational memory were significantly impaired. Brain MRI was normal. The de novo heterozygous c.326_327insA or c.326dupA, p.(Ser110Valfs*2) loss-of-function variant in *NR4A2* is predicted to cause a premature stop codon leading to either nonsense-mediated decay or shortened nonfunctional protein [135].

#### 3.3.6. *RAB39B*

RAB39B (Ras-related protein Rab-39B), a GTPase of the RAB family, whose members play an essential role in intracellular vesicular trafficking, is a neuronal-specific protein localizing to the Golgi compartment. One of its functions is to drive glutamate ionotropic receptor AMPA type subunit 2/3 (GluA2/GluA3) maturation and trafficking. RAB39B knock-out mice show immature spine arrangement and behavioral and cognitive abnormalities, which derive from altered neuronal dendritic spine refinement, resulting in a more calcium (Ca^2+^)-permeable and excitable synaptic network [136,137]. However, the full impact of *RAB39B* loss of function on synaptic activity is still largely unexplained. 

Pathogenic variants in the *RAB39B* gene cause X-linked ID with ASD and epilepsy, also comorbid with early-onset PD [138]. A few patients have been reported on with ID, delayed or even normal early motor milestones, language delay, macrocephaly, and early-onset Parkinsonism. ASD was present in 2/6 in the D-23 kindred [139] and 1/8 in the MRX72 family. Epilepsy was reported in three brothers in the MRX72 family, with onset at 3, 6 and 9 years of age, respectively [140], and in 2/6 brothers in the Wisconsin pedigree, with onset in early childhood (2 years of age in the first brother, not reported in the second) [141]. In the Wisconsin pedigree, six affected brothers were longitudinally evaluated over a 15-year period. An additional six relatives were suspected of being affected. They had varying degrees of global DD and subsequent ID, varying degrees of megalencephaly, and an extrapyramidal syndrome (cogwheel rigidity, resting tremor, and postural changes) during their second decade of life, although the most severely affected brother already had tremors, choreoathethoid movements, and upper limbs rigidity when first examined at 10 years of age. They had hypokinesia and hypokinetic dysarthria. Their gait was slow with poor balance and some showed a shuffling gait (no pyramidal signs). Three patients received a trial of levodopa, withdrawn in all, due to side effects in one and to inefficacy in the remaining two. Two brothers had epilepsy, with onset in early childhood, which was difficult to control in one, while an additional two brothers had mildly abnormal EEGs [141]. 

A shuffling gait was also reported in 2/3 patients in the Australian kindred, who had an akinetic-rigid syndrome. In this kindred, the age range at tremor onset was wider than in the Wisconsin kindred (from late childhood to 44 years), and a diagnosis of PD was made at 44 and 45 years in 2/3 patients, while the third did not show any disease progression. Unlike the Wisconsin family, response to levodopa was favorable [138].

#### 3.3.7. *MECP2* Pathogenic Variant p.(Ala140Val) in Males

*MECP2*-related disorders in males include severe neonatal encephalopathy (OMIM # 300673), intellectual developmental disorder 13 (MRXS13) (OMIM # 300055), pyramidal signs, Parkinsonism and macroorchidism (PPMX) (OMIM # 300055), Rett syndrome classical phenotype (OMIM #312750), and *MECP2* duplication syndrome (OMIM #300260) [142].

A recent systematic review focusing on male patients carrying pathogenic or likely pathogenic variants in the *MECP2* gene documented 49 cases (46.2%) of MRXS13, 32 cases (30.2%) of severe neonatal encephalopathy, and 25 cases (23.6%) of PPMX. 

So far, MRXS13 has been reported in 25 families with 47 individuals carrying 21 different pathogenic variants, usually located at the final 3′ end of the *MECP2* gene and mainly leading to premature stop or in-frame deletion. Some missense variants (not causative of Rett syndrome in females) can also lead to non-syndromic ID in males. Mild-to-severe DD or ID and epilepsy were reported in 16/46 patients. Mood and behavioral problems occur in a subset of affected individuals [143]. PPMX is a specific phenotype documented in males and females carrying the p.(Ala140Val) pathogenic variant. It is characterized by DD/ID with juvenile loss of previously acquired skills and motor deterioration, with onset of Parkinsonism and pyramidal signs. Macroorchidism (detected in nine cases) and psychiatric manifestations (mainly psychosis, detected in five cases) are seen in some. Importantly, none of the patients in this group so far presented with epilepsy. Patients’ age at the time of report ranged from 11 to 67 years [143]. The authors did not record any overlapping phenotypes between MRXS13 and PPMX. However, as epilepsy is not present in all of the MRXS13 patients, longer follow-up would be useful to detect the possible development of extrapyramidal and/or pyramidal signs in these patients (Table 4).

## 4. Discussion

The main monogenic and copy number variation causing pediatric-onset epilepsies or DEE with early-onset Parkinsonism were reviewed. Clinically, a typical lifespan evolution of phenotypes, as seen in many childhood-onset MDs, can be confirmed: from infantile hypotonia to hyperkinetic MD in childhood, followed by rigidity and hypokinesia in teenage and juvenile years [144]. 

A few discrete presentations can be identified: Parkinsonism developing as a late manifestation of complex NDD, characterized by DE-EE (usually with multiple, refractory seizure types and severely abnormal background EEG), with or without a preceding hyperkinetic MD; Parkinsonism in the context of syndromic conditions in which an unspecific reduced seizure threshold is present in infancy and childhood; neurodegenerative conditions with brain iron accumulation, in which childhood DE-EE is followed by neurodegeneration; and finally monogenic juvenile Parkinsonism, in which a subset of patients develop signs and symptoms of a hypokinetic MD from the second to fourth decade of life, usually in the context of DD/ID, after a childhood marked by unspecific and usually well controlled epilepsy.

From a functional point of view, the role of the causative genes in synaptic vesicle formation, trafficking and recycling («synaptopathies»), autophagy and lysosomal degradation, ER stress/dysfunction, and mitochondrial dysfunction potentially links the pathogenesis of these disorders to acknowledged patho-mechanisms of PD. 

In detail, synaptic vesicle cycling (SVC) genes control neurotransmitter release and recycling, and are critical for synaptic transmission and plasticity. They are all recognized as important biological pathways in the aetiology of epilepsy with NDD [124], serving as the pathophysiological basis for developing complex neurological phenotypes (ID/DD, epilepsy, MDs, and possibly psychiatric comorbidities), which are frequent comorbidities. Seizure semiology varies widely, and different explanations can be hypothesized. For example, epilepsy-associated variants may have a stronger impact on early neurite development (i.e., *STXBP1* [145], *TBC1D24* [52], *SYNJ1* [146]), while variants impacting only on post-embryological SVC formation [145] might determine lower epilepsy risk. Alternatively, affected sub-processes might result in phenotypic differences. The highest epilepsy frequency is seen in the docking and priming subgroup (*STXBP1*), and the lowest in the clustering group. The involvement in trans- versus post-synaptic zone, cell-specific gene expression, variant-specific effects, and modifying factors might also account for these differences [144]. Disorders of clustering and scaffolding have the lowest risk of MD. Disorders of docking and priming (i.e., *STXBP1*) are rarely associated with severe dystonia and dyskinesia, but commonly with tremor and ataxia. Thus, disturbances to different aspects of SVC might have a different impact on different neuronal systems involved in motor control [144]. The concept of synaptopathy is closely related to α-synuclein, which is primarily localized to the presynaptic terminals, where it affects fusion and clustering of synaptic vesicles, influencing neurotransmitter release [146,147,148,149]. *STXBP1* plays a key role in chaperoning α-synuclein, and its deletion promotes α-synuclein aggregation [29], potentially explaining the neurodegenerative course observed since adolescence in patients with *STXBP1*-related DEE, and in line with animal knockdown studies [32]. Furthermore, different AR missense and truncating variants in the *SYNJ1* gene have been linked to both early and later onset neurodegeneration, suggesting a key role of presynaptic vesicle proteins in neuronal survival [32].

Autophagy, acting through lysosomal degradation, represents the main proteolytic system in neurons [150]. Abnormal autophagy leads to accumulation of aberrant proteins and toxic components [151], and is considered as the main patho-mechanism leading to the neurodegenerative phase of BPAN, but also contributing to PD [152], Alzheimer’s and HD [153].

Mitochondrial dysfunction caused by bioenergetic defects, pathogenic variants in mitochondrial DNA or nuclear DNA genes, impaired transcription, changes in mitochondrial dynamics or morphology, impaired trafficking or transport are all implicated in PD pathogenesis [5]. Deficiencies in complexes of the respiratory chain, suggesting secondary mitochondrial impairment, have been documented anecdotally in one *STXBP1* patient [30], but complex I and IV deficiency has also been documented in sporadic Parkinson’s disease [154]. 

The regional distribution of gene products at both cortical and subcortical levels can be regarded as a clue to the pathogenesis of comorbid MDs and epilepsy [155], possibly with different neuronal vulnerability to specific patho-mechanisms, explaining differences in penetrance. Age-dependent differences in the predominant site of expression of gene products within brain regions might explain the shift between epilepsy or DE-EE to later neurodegenerative phase. For example, *FOXG1* initially plays an important role in the development of fetal telencephalon, being especially expressed in the anterior brain, while in adults it is highly expressed in basal ganglia. Similarly, while in embryonic mice *FRRS1L* is expressed in the forebrain [59], in adult mice its expression is high in the cerebral cortex, hippocampus, midbrain, striatum, granule cells of the dentate gyrus and Purkinje cells [69], being upregulated during postnatal development [67]. Dopaminergic dysfunction is an additional mechanism, and the known vulnerability of the dopaminergic system to ageing can explain its age of onset [51]. 

However, when evaluating rare and complex neurological phenotypes, it is possibly difficult to distinguish between phenotypic variability, age-dependency of symptoms, and underlying genetic modifiers. In patients with 22q11.2 deletion syndrome, whole genome sequencing was used to investigate sequence variants in genes associated with increased PD risk [85]. Such an approach might prove useful in other conditions, especially whenever only anecdotal reports exist.

Future clinical studies should focus on accurate phenotypic and genotypic description of large cohorts of patients, with long-term follow-up, and consistently report on response to drug therapy for both epilepsy and MD. Whole genome-based studies might be required in order to fully understand the role of additional sequence variants in susceptibility genes for MDs and especially Parkinsonism. 

## 5. Conclusions

In conclusion, an emerging group of genetic conditions leading to epilepsy or EE in childhood followed by juvenile Parkinsonism highlights the need for careful long-term follow-up, especially in the context of DD or ID, in order to readily identify individuals at increased risk of later Parkinsonism.

## Figures and Tables

**Table 2:** Chromosomopathies and copy number variations with increased risk of epilepsy and Parkinsonism.

Condition Name	Epilepsy	Parkinsonism	Additional Developmental and Psychiatric Features
22q11.2 deletion syndrome	Increased risk of seizures (provoked, unprovoked, focal or generalized)	Teenage to adult years	Psychiatric symptoms, ADHD, ASD, ID
6q26–q27 Deletion	Increased risk of (drug-responsive) seizures	Single report	ID, ASD, ADHD
Trisomy 21	Increased risk of epilepsy, West syndrome	EOP	ID, ASD, ADHD, increased risk of dementia
Klinefelter syndrome	Increased risk of epilepsy	Single report (27 y)	ID
Partial 6q syndrome	Not known to be associated with increased risk of epilepsy	Single report (35 y)	ID
Partial 4q syndrome	Not known to be associated with increased risk of epilepsy	Single report (30 y)	ID

**Table 3:** Neurodegenerative disorders possibly presenting with epilepsy followed by Parkinsonism.

	Inheritance	Epilepsy	Parkinsonism	Additional Features
*WDR45*	XL	Childhood	Early adulthood	DD, regression, cognitive decline
*IT15*	CAG repeats expansion	Childhood	Childhood	Aggressive, hyperactive, oppositional behavior

**Table 4:** Juvenile Parkinsonism genes with possible association of epilepsy.

	Inheritance	Epilepsy, Age at Onset	Parkinsonism, Age at Onset	Developmental and Additional Neurological Features
*KCND3*	AD	Infancy-childhood (early-onset phenotype—see text)	Second to fifth decade	Ataxia, oculomotor disorders, possible ID, NDD, myoclonus, dystonia, tremor, peripheral neuropathy, pyramidal signs
*ATP6AP2*	XL	Infancy	First to sixth decade	Mild-to-moderate ID, ataxia, scholiosis
*DNAJC6*	AR	1–5 y	First to third decade	Early-onset tremor, shuffling gait, and/or bradykinesia
*SYNJ1*		Infancy to adolescence	Second to third decade	Mild cognitive impairment
*NR4A2*	AD	Childhood to early adulthood	Infantile-onset to early-onset parkinsonism	Poor feeding, gastrointestinal symptoms, normal motor but delayed speech development
*RAB39B*	XL	Early childhood	Second to fifth decade	ID, ASD, macrocephaly, tremors, choreoathethoid movements and upper limbs rigidity (10 y)
*MECP2* pathogenic variants in males	XL	Possible (with childhood onset) in XLMR13 phenotype; absent in PPM-X phenotype	After the first decade (PPM-X phenotype)	Mild-to-severe DD or ID, mood and behavioral problems, psychosis

## Data Availability

Data supporting reported results can be found in the original papers.
